# Not Always a Walk in the Park: Evaluating Trends and Disparities in Community Mobility Among Older Adults, 2015-2022

**DOI:** 10.1093/geroni/igaf122.3362

**Published:** 2025-12-31

**Authors:** Jason Falvey, Chixiang Chen, Michelle Shardell, Jay Magaziner, C Daniel Mullins

**Affiliations:** University of Maryland School of Medicine, Baltimore, Maryland, United States; University of Maryland School of Medicine, Baltimore, Maryland, United States; University of Maryland School of Medicine, Baltimore, Maryland, United States; University of Maryland School of Medicine, Baltimore, Maryland, United States; University of Maryland School of Pharmacy, Baltimore, Maryland, United States

## Abstract

Maintaining the ability to navigate community distances, or at least three blocks (approximately ¼ mile), is crucial for older adults. Inability to do so is associated with diminished well-being. Our sample included community-dwelling participants aged 70 years and older from the National Health and Aging Trends Study, an ongoing nationally representative annual survey of older Medicare beneficiaries Community mobility was defined as the capacity to walk 3+ blocks without assistance. Repeated cross-sectional trends analysis (SAS-callable SUDAAN) and survey-weighted logistic regression was used to evaluate age, sex, and race and ethnicity–stratified proportions of older adults who could walk at least 3 blocks in their community and changes in this rate over time. Overall, 28% of older adults (or an estimated 7.5 million) could not walk three blocks in 2015; 8.8 million older adults (25%) were unable to walk three blocks in 2022. Consistently, Black and Hispanic participants had significantly lower rates of community mobility than White participants (62% and 63% vs 77% by 2022, respectively; all years p<.0001). Female participants also demonstrated persistently lower rates. Hispanic older adults experienced a notable decline in community mobility rates from 2015 (70%) to 2022 (63%), and modest improvements were observed for Black older adults (58% to 62%). Overall, more than 8 million older adults in the US may have difficulty walking 3 blocks outside, which has only modestly improved over the past 7 years. These findings highlight the need for targeted interventions to address limited and inequitable community mobility in the US.

